# Linking Structure to Dynamics in Protic Ionic Liquids: A Neutron Scattering Study of Correlated and Single-Particle Motions

**DOI:** 10.1038/s41598-018-34481-w

**Published:** 2018-11-06

**Authors:** Tatsiana Burankova, Juan F. Mora Cardozo, Daniel Rauber, Andrew Wildes, Jan P. Embs

**Affiliations:** 1 Laboratory for Neutron Scattering and Imaging, Paul Scherrer Institute, 5232 Villigen, PSI Switzerland; 2 Department of Physical Chemistry, Saarland University, 66123 Saarbrücken, Germany; 3 Institut Laue-Langevin, CS 20156, 38042 Grenoble Cedex 9, France

## Abstract

Coupling between dynamical heterogeneity of ionic liquids and their structural periodicity on different length-scales can be directly probed by quasielastic neutron scattering with polarization analysis. The technique provides the tools to investigate single-particle and cooperative ion motions separately and, thus, dynamics of ion associations affecting the net charge transport can be experimentally explored. The focus of this study is the structure-dynamic relationship in the protic ionic liquid, triethylammonium triflate, characterized by strong hydrogen bonds between cations and anions. The site-selective deuterium/hydrogen-isotope substitution was applied to modulate the relative contributions of different atom groups to the total coherent and incoherent scattering signal. This approach in combination with molecular dynamics simulations allowed us to obtain a sophisticated description of cation self-diffusion and confined ion pair dynamics from the incoherent spectral component by using the acidic proton as a tagged particle. The coherent contribution of the neutron spectra demonstrated substantial ion association leading to collective ion migration that preserves charge alteration on picosecond time scale, as well as correlation of the localized dynamics occurring between adjacent ions.

## Introduction

Understanding dynamical and structural heterogeneity of ionic liquids (ILs)^1–3^ has important implications for explaining and predicting their macroscopic physico-chemical properties (viscosity, thermal and electrical conductivity, melting points, etc) relevant for diverse applications^4,5^. Numerous X-ray and neutron diffraction studies as well as computer simulations have already provided a clear picture of the long-range and intermediate-range order in ILs, which distinguishes these novel materials from conventional solvents^2,3,6^. Dynamics on various time scales have been characterized by many spectroscopic techniques such as different types of NMR^7–9^, dielectric and optical Kerr effect^10^, and time-resolved fluorescence^11^ spectroscopies. In this perspective, quasielastic neutron scattering (QENS)^12^ is a unique technique, because it can simultaneously probe both structure and dynamics and, hence, enables a direct observation of structure-dynamics relationships in ILs. Due to the large neutron incoherent cross section of hydrogen, the method is predominantly sensitive to single-particle (uncorrelated) motions of protons ubiquitous in organic cations of ILs^13–16^. Selective deuterium/hydrogen (D/H) isotope substitution can be applied to intensify coherent scattering^14,17,18^ and to shift the research focus to collective (correlated) processes governed by the charge alteration in ILs^19,20^.

The full wealth of available information hidden in both incoherent and coherent parts of a QENS spectrum is, however, difficult to extract. The knowledge about the composition of the sample and the corresponding neutron cross section is not sufficient to disentangle collective and single particle processes. The only unambiguous experimental way to overcome this problem is to perform QENS measurements in conjunction with polarization analysis. This approach is getting more common in soft matter research^21–23^ and has also appeared to be successful in the case of pyridinium-based ILs^22^. The separation of coherent and nuclear spin-incoherent scattering clearly demonstrated a collective nature of the long-range diffusion of butyl-pyridinium cations determined by Coulomb and Van der Waals forces between ions, whereas the localized dynamics of the long alkyl chain turned out to be a true single-particle process. Changing the balance between the ion interactions by introducing highly directional hydrogen bonds, as in the case of protic ionic liquids (PILs) produced by combining a Brønsted acid and a Brønsted base^1^, may lead to additional coherence effects in ion dynamics. Therefore, QENS with polarization analysis can be seen as a valuable and informative method for understanding microscopic details of hydrogen bonding and proton conductivity in such systems.

During the last years, several publications on single-particle dynamics in PILs have appeared in the literature^14–16^. In general, the PIL spectra are interpreted in an analogous way as those of more extensively studied aprotic ILs^17,18,24,25^, assuming long-range diffusion and various subdiffusive (localized) motions. As acidic protons can hypothetically contribute to anhydrous conductivity of PILs via Grotthus mechanism (proton hopping through the hydrogen bond network), much attention is also explicitly paid to its dynamics. For example, comparing [Im][TFSI] and its deuterated analogue [dIm][TFSI], Hoarfrost *et al*.^15^ analyzed the temperature dependent efficiency of proton transfer events. In our recent work^14^ we investigated triethylammonium triflate (TEA-TF, or [NH(C_2_H_5_)_3_][SO_3_CF_3_]) with the prevailing vehicular mechanism of the acidic proton transport as it has been shown by pulsed field gradient NMR (PFG-NMR)^26,27^ and also proved by our computer simulations^28^. Using D/H-isotope substitution in the ethyl chains of the cation we left the proton attached to the triethylamine unchanged to be used as a tagged particle. The most interesting finding was an additional localized process of this N-H proton on the time scale of ~4 ps even in the solid phase, when the global motions of the cations are “frozen”. However, the obtained experimental data were not sufficient to unambiguously determine the nature of this process, first of all, due to the presence of the relatively strong coherent contribution of the partially deuterated sample. Therefore, an experimental separation of coherent and nuclear spin-incoherent scattering was required for a more sophisticated analysis.

The current work is a combined experimental and computational study on TEA-TF. Here we present QENS experiments with polarization analysis on both completely protonated and partially deuterated (TEA_D_-TF, [NH(C_2_D_5_)_3_][SO_3_CF_3_]) samples in the time window of tens picoseconds. We aim not only to refine the previous results by excluding coherence effects, but also to provide a complete and thorough picture of both single-particle and collective motions in this model PIL, and in this way to show the relationship between the structure and dynamics. Taking into account the complexity of the system, its structural and dynamical hierarchy, it is essential for interpreting experimental data to use input from the molecular dynamics (MD) simulations^24^, which cover approximately the same time and distance scale as QENS. The computational support is especially valuable in the case of the collective dynamics, where cation-cation, anion-anion and cation-anion correlated motions give origin to the coherent signal.

## Data Analysis

As it follows from the theoretical principles of the polarization analysis^29,30^, the experimental scattering intensity can be separated into the coherent and nuclear spin-incoherent contributions, which are directly related to the corresponding dynamic structure factors, ${S}_{{\rm{coh}}}(Q,E)$ and ${S}_{{\rm{inc}}}(Q,E)$, defined as Fourier transforms of the intermediate scattering functions, ${I}_{{\rm{coh}}}(Q,t)$ and ${I}_{{\rm{inc}}}(Q,t)$:

1 $${S}_{{\rm{coh}}/{\rm{inc}}}(Q,E)=\frac{1}{2\pi \hslash }\,{\int }_{-\infty }^{\infty }\,{I}_{{\rm{coh}}/{\rm{inc}}}(Q,t)\,\exp \,(\,-i\frac{E}{\hslash }t)\,dt$$

The next expressions demonstrate the connection between the intermediate scattering functions and the position operators in a system of identical particles:

2a $${I}_{{\rm{coh}}}(Q,t)=\frac{1}{N}\,\sum _{i,j}\,\langle {{\rm{e}}}^{-i{\bf{Q}}{{\bf{r}}}_{i}\mathrm{(0)}}{{\rm{e}}}^{i{\bf{Q}}{{\bf{r}}}_{j}(t)}\rangle $$

2b $${I}_{{\rm{inc}}}(Q,t)=\frac{1}{N}\,\sum _{i}\,\langle {{\rm{e}}}^{-i{\bf{Q}}{{\bf{r}}}_{i}\mathrm{(0)}}{{\rm{e}}}^{i{\bf{Q}}{{\bf{r}}}_{i}(t)}\rangle $$

where *N* is the total number of scatterers in the system; the angular brackets $\langle \cdots \rangle$ denote a thermodynamic average. The coherent contribution is determined by the correlation between the positions of different nuclei (*i*, *j*) at different times and originates from interference effects, while the incoherent contribution provides information on single-particle relaxations. In the case of different types of nuclei the intermediate scattering functions have to be averaged with weights depending on their neutron scattering lengths, *b*_*i*_:

3a $${I}_{{\rm{coh}}}(Q,t)\sim \sum _{i,j}\,{b}_{i,{\rm{coh}}}{b}_{j,{\rm{coh}}}\,\langle {{\rm{e}}}^{-i{\bf{Q}}{{\bf{r}}}_{i}\mathrm{(0)}}{{\rm{e}}}^{i{\bf{Q}}{{\bf{r}}}_{j}(t)}\rangle $$

3b $${I}_{{\rm{inc}}}(Q,t)\sim \sum _{i}\,{b}_{i,{\rm{inc}}}^{2}\,\langle {{\rm{e}}}^{-i{\bf{Q}}{{\bf{r}}}_{i}\mathrm{(0)}}{{\rm{e}}}^{i{\bf{Q}}{{\bf{r}}}_{i}(t)}\rangle $$

These formulas enable calculation of neutron spectra from MD trajectories^31^ and, hence, a direct comparison with the experimental scattering functions.

The model description of cation single-particle dynamics on a picosecond time scale generally implies a superposition of two relaxation processes^14,17,18^. The first one is the long-range diffusion, the second one comprises entangled localized motions of side groups (conformational changes of alkyl groups, librations). To resolve the third subpicosecond component reported for ILs^13,18,25^, the probed dynamical range has to be expanded by decreasing the wavelength of incident neutrons. With the applied experimental setting in the present study the fast relaxation is detected only as a flat background contribution. Because the characteristic linewidths of the confined and long-range processes differ by approximately a factor of ten, the incoherent dynamic structure factor can be presented as a convolution of the independent global, ${S}_{{\rm{glob}}}(Q,E)$, and localized, ${S}_{{\rm{loc}}}(Q,E)$, dynamic structure factors, multiplied by a Debye–Waller factor, $\exp (\,-\,2W)$.

4 $${S}_{{\rm{inc}}}(Q,E)=\exp (\,-\,2W){S}_{{\rm{glob}}}(Q,E)\otimes {S}_{{\rm{loc}}}(Q,E)$$

The adequate modeling of the long-range process in ILs is based on the jump-diffusion model^32^, where ${S}_{{\rm{glob}}}(Q,E)$ has a Lorentzian shape with the half-width ${{\rm{\Gamma }}}_{{\rm{tr}}}$ depending on *Q* as follows:

5 $${{\rm{\Gamma }}}_{{\rm{tr}}}(Q)=\frac{\hslash {D}_{{\rm{tr}}}{Q}^{2}}{1+{D}_{{\rm{tr}}}{Q}^{2}{\tau }_{0}}$$

where *D*_tr_ is the self-diffusion coefficient and ${\tau }_{0}$ is the residence time. It is necessary to mention that, because the time scale of the discussed QENS measurements does not exceed tens of picoseconds, *D*_tr_ has the meaning of a short-time diffusion constant. Experiments in a broader time window clearly show that the formalism of the jump-diffusion model is not strictly valid and the so-called stretched exponential function is required to characterize the long-range process^13,18,25^. For this reason diffusion coefficients evaluated by other methods such as PFG-NMR^26,27^ are usually smaller than those obtained from QENS experiment.

To describe various localized cation motions we applied the Gaussian model^33^, which considers particles moving inside a confinement with a “soft” boundary.

6 $${S}^{{\rm{G}}}(Q,E;{D}_{{\rm{loc}}},R)={{\rm{e}}}^{-{Q}^{2}{R}^{2}}[\delta (E)+\sum _{n=1}^{\infty }\,\tfrac{{({Q}^{2}{R}^{2})}^{n}}{n!}\tfrac{1}{\pi }\tfrac{\hslash n{D}_{{\rm{loc}}}/{R}^{2}}{{(\hslash n{D}_{{\rm{loc}}}/{R}^{2})}^{2}+{E}^{2}}]$$

where *D*_loc_ stands for the self-diffusion coefficient of the localized motion and *R* is the variance of the particle displacement and characterizes the size of the domain, in which the particles are diffusing. Taking into account that the incoherent dynamic structure factor does not contain any cross-correlation terms and, hence, is an additive function, ${S}_{{\rm{loc}}}(Q,E)$ for the whole cation can be given as a simple sum of the three terms accounting for the motions of “equivalent” hydrogens diffusing in a confinement with the corresponding characteristic radius (*R*_H_, *R*_1_, and *R*_2_ for the N-H proton, bridging methylene, and end methyl groups, respectively). The contribution of the other elements (C, N, S, O) can be neglected (Table S1 of the Supporting Information). The three terms of ${S}_{{\rm{loc}}}(Q,E)$ have to be weighted with respect to the total number of particles in each group and the incoherent neutron cross sections of H and D.

7a $${S}_{{\rm{loc}}}^{{\rm{prot}}}(Q,E)=\tfrac{1}{16}{S}_{{\rm{H}}}^{{\rm{G}}}(Q,E;{D}_{{\rm{H}}},{R}_{{\rm{H}}})+\tfrac{6}{16}{S}_{{\rm{1}}}^{{\rm{G}}}(Q,E;{D}_{{\rm{ch}}},{R}_{1})+\tfrac{9}{16}{S}_{{\rm{2}}}^{{\rm{G}}}(Q,E;{D}_{{\rm{ch}}},{R}_{2})$$

7b $$\begin{array}{rcl}{S}_{{\rm{loc}}}^{{\rm{deut}}}(Q,E) & = & \tfrac{{\sigma }_{{\rm{H}}}}{15{\sigma }_{{\rm{D}}}+{\sigma }_{{\rm{H}}}}{S}_{{\rm{H}}}^{{\rm{G}}}(Q,E;{D}_{{\rm{H}}},{R}_{{\rm{H}}})+\tfrac{6{\sigma }_{{\rm{D}}}}{15{\sigma }_{{\rm{D}}}+{\sigma }_{{\rm{H}}}}{S}_{{\rm{1}}}^{{\rm{G}}}(Q,E;{D}_{{\rm{ch}}},{R}_{1})\\  &  & +\,\tfrac{9{\sigma }_{{\rm{D}}}}{15{\sigma }_{{\rm{D}}}+{\sigma }_{{\rm{H}}}}{S}_{{\rm{2}}}^{{\rm{G}}}(Q,E;{D}_{{\rm{ch}}},{R}_{2})\end{array}$$

where the superscripts “prot” and “deut” are used for the TEA-TF and TEA_D_-TF samples, *σ*_H_ and *σ*_D_ are the neutron incoherent cross sections of the hydrogen isotopes H and D, respectively. The diffusion coefficients for the hydrogens of the ethyl groups are considered to be equal to each other (*D*_ch_). Although it is obvious that the flexibility of the alkyl groups may result in a distribution of both radii of confinement and diffusion coefficients, as it is suggested, for example, from MD simulations, we had to apply this approximation to ensure the stability of the fit parameters.

Finally, the model dynamic incoherent structure factor (Eq. 4 including Eqs 5–7) convoluted with the resolution function of the instrument, *R*(*Q*, *E*), is fitted to the measured scattering intensity:

8 $${I}_{{\rm{m}}{\rm{e}}{\rm{a}}{\rm{s}}}\sim {S}_{{\rm{i}}{\rm{n}}{\rm{c}}}(Q,E)\otimes R(Q,E)+bg(Q)$$

where *bg*(*Q*) is a flat background accounting for faster relaxations unresolved in the accessible experimental time window.

The fits of a pair of the TEA-TF and TEA_D_-TF spectra were performed in an iterative way at each measured temperature point. In the initial parameter set for the TEA-TF sample, the contribution of the acidic proton was neglected. This provided the first estimates for the other parameters *D*_tr_, *D*_ch_, *R*_1_, and *R*_2_, which were used in the next step of fitting of the TEA_D_-TF spectrum. The procedure was repeated until the difference in the parameters values of two successive iterations was significantly less than the error margins (5-6 times). Examples of the incoherent spectra fitted with the model scattering function are presented in Fig. S7 of the Supplementary Information.

The number of known analytical models applicable for coherent scattering is significantly smaller than that for the simpler case of incoherent scattering. For example, correlated reorientational motions can be characterized in the systems of non-interacting identical ions/molecules^34,35^. There are also examples of more general descriptions such as Vineyard’s static approximation^36^ and Sköld’s ad-hoc ansatz^37^. However, these approaches cannot be directly and unambiguously transferred to the coherent scattering of ILs, where different types of both intramolecular and intermolecular correlations lead to the appearance of diffraction peaks^2,3^ in the Q-range accessible by QENS and have impact on dynamics^19,22^. For this reason, in the present work we will use a model-independent approach assuming that the total coherent dynamic structure factor is a convolution of the correlated long-range (subscript tr) and localized (subscript loc) relaxation processes:

9 $${S}_{{\rm{coh}}}(Q,E)=S(Q)\,\exp (\,-\,2W)\tfrac{1}{\pi }\tfrac{{{\rm{\Gamma }}}_{{\rm{tr}}}^{{\rm{coh}}}(Q)}{{{\rm{\Gamma }}}_{{\rm{tr}}}^{{\rm{coh}}}{(Q)}^{2}+{E}^{2}}\otimes [A(Q)\delta (E)+\mathrm{(1}-A(Q))\tfrac{{{\rm{\Gamma }}}_{{\rm{loc}}}^{{\rm{coh}}}(Q)}{{{\rm{\Gamma }}}_{{\rm{loc}}}^{{\rm{coh}}}{(Q)}^{2}+{E}^{2}}]$$

where S(Q) is the coherent structure factor, the other parameters ${{\rm{\Gamma }}}_{{\rm{tr}}}^{{\rm{coh}}}(Q)$, ${{\rm{\Gamma }}}_{{\rm{loc}}}^{{\rm{coh}}}(Q)$ and *A*(*Q*) are modulated by S(Q) and do not have an explicit analytical description. Examples of the coherent spectra fitted with the model scattering function are presented in Fig. S8 of the Supplementary Information. The qualitative picture of correlated motions in TEA-TF provided by Eq. 9 will be interpreted in terms of anion-anion, cation-anion, and cation-cation contributions (Eq. 3) with the help of the MD simulations.

## Results and Discussion

### Diffraction with Polarisation Analysis

**Figure 1:**
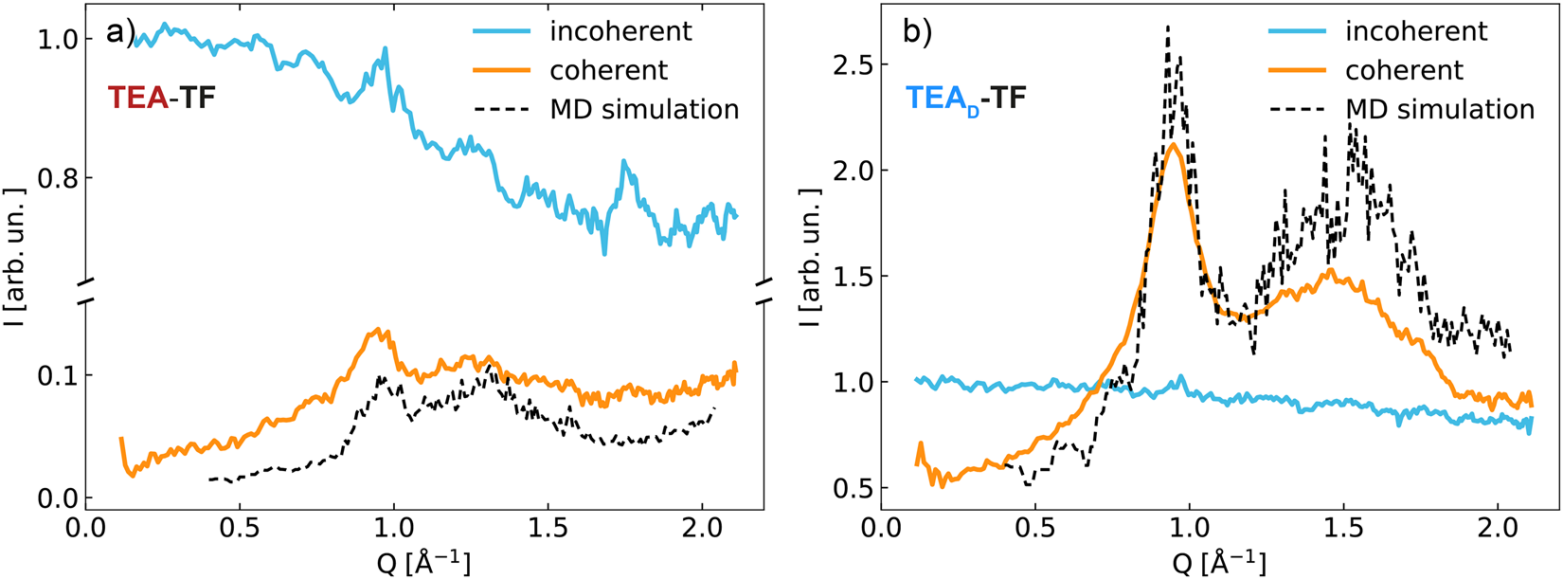
Coherent (orange line) and incoherent (blue line) contributions of the diffraction spectrum of TEA-TF (**a**) and TEA_D_-TF (**b**) measured at T = 320 K. The experimental data are compared to the cross-section weighted structure factor calculated from the MD particle configuration (black dashed line). The component intensities are normalized to ${I}_{{\rm{inc}}}(Q\to 0)$ for clarity.

Using the D7 diffuse scattering spectrometer in the diffraction mode we determined the nuclear spin-incoherent and coherent parts of the diffraction pattern as presented in Fig. 1. The nuclear spin-incoherent scattering dominates the TEA-TF spectrum, whereas the coherent signal of the partially deuterated TEA_D_-TF is comparable and even stronger than the self-correlation contribution in the probed Q-range. Energy redistribution due to high-frequency vibrations (Debye-Waller factor) leads to a gradual decay of the incoherent component with Q. The mean square displacement (msd, $\langle {u}^{2}\rangle$) associated with these vibrations can be estimated from the formula $\exp (\,-\,2W)=\exp (\,-\,\langle {u}^{2}\rangle {Q}^{2})$. The fast vibrational motions of the ethyl protons significantly change the total msd of TEA-TF ($\langle {u}^{2}\rangle$ = 0.12 Å^2^ at T = 320 K) in comparison to TEA_D_-TF ($\langle {u}^{2}\rangle$ = 0.05 Å^2^ at T = 320 K).

**Figure 2:**
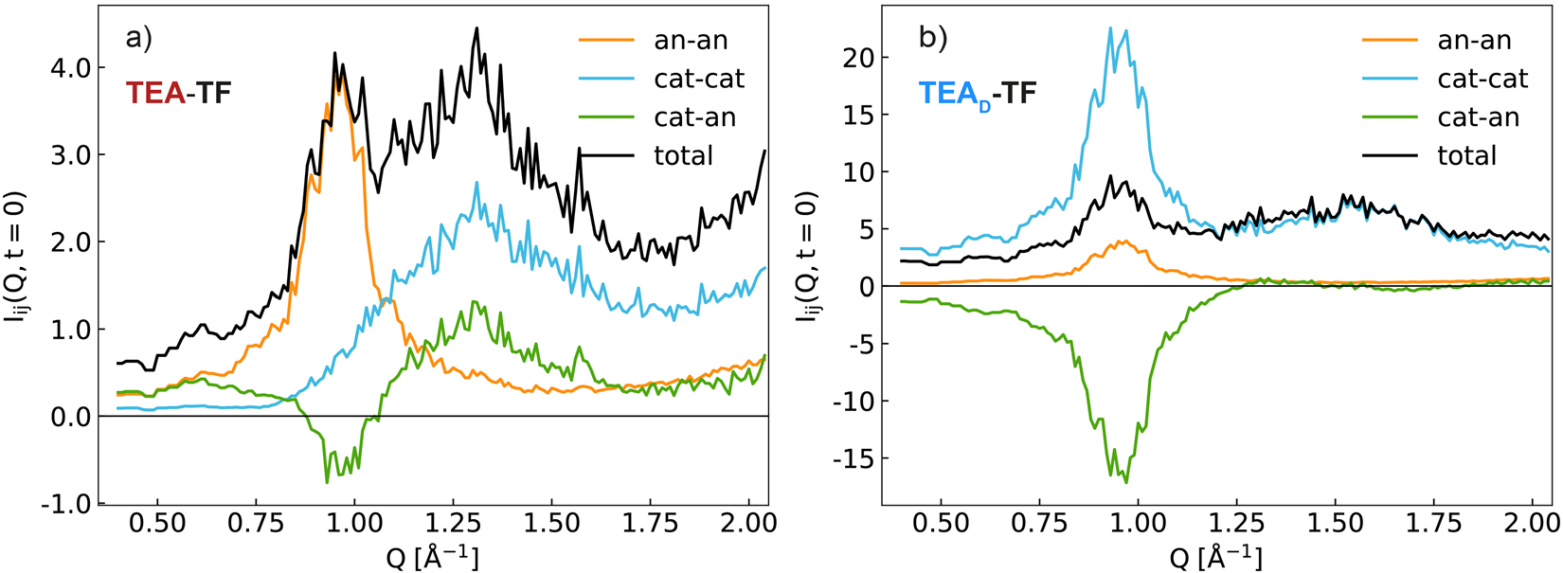
Anion-anion (orange), cation-cation (blue) and cation-anion (green) subcomponents of I(Q, t = 0) for TEA-TF (**a**) and TEA_D_-TF (**b**) as obtained from the MD particle configuration at T = 320 K. The subcomponent intensities are determined by the neutron coherent scattering lengths of the species and their total number as given in Eq. 3b.

The coherent part of the diffraction spectra exhibits a pattern typical for other ILs^2,3^ with two correlation peaks at about 0.9 Å^−1^ and 1.3–1.8 Å^−1^. The latter is the so-called adjacency peak^3^, the position of its maximum shifts with the deuteration of the ethyl chain pointing out to the intramolecular as well as intermolecular origin of the feature. The low-Q peak at 0.9 Å^−1^ is a signature of the unique charge ordering in ILs and referred to as the charge-charge correlation peak. Its position is not influenced by the isotope substitution in the cations. The data are compared with $S(Q)={I}_{{\rm{coh}}}(Q,0)$ calculated from the MD trajectories using Eq. 3. The experimental and theoretical results show a relatively good agreement, especially for the peak positions, allowing us to use the results of the MD analysis in the interpretation of the experimentally observed dynamics. For example, it is possible to dissect contributions of different atom groups and construct anion-anion (an-an), cation-cation (cat-cat) and cation-anion (cat-an) subcomponents of *S*(*Q*) by adding up corresponding cross-section weighted terms in Eq. 3 (Fig. 2).

All the subcomponents exhibit intense peaks or antipeaks at 0.9 Å^−1^. This picture is typical for systems of two species distributed with equal periodicity^3^. The adjacency correlation peak at 1.3–1.8 Å^−1^ of the neutron spectrum is mainly formed by the cation cross-correlation functions in both TEA-TF and TEA_D_-TF. The main difference is that the carbon skeleton contributes largely to the total S(Q) of the protonated sample, whereas the deuterium atoms are responsible for the dominating part of S(Q) in TEA_D_-TF.

### Single Particle Dynamics

Long-range translation, localized conformational and librational motions of the ethyl chains as well as the restricted dynamics of the acidic proton have been previously described on a picosecond time scale in TEA-TF^14^. The interpretation of the results has been, however, based on the assumption that any interference effects of the inter- and intramolecular processes relax fast enough and do not distort the incoherent signal originating from the localized ion motions, as it was previously observed for a pyridinium-based IL^22^. Experimental separation of coherent and nuclear spin-incoherent scattering allows us to estimate the limits of validity of this approach for TEA-TF.

**Figure 3:**
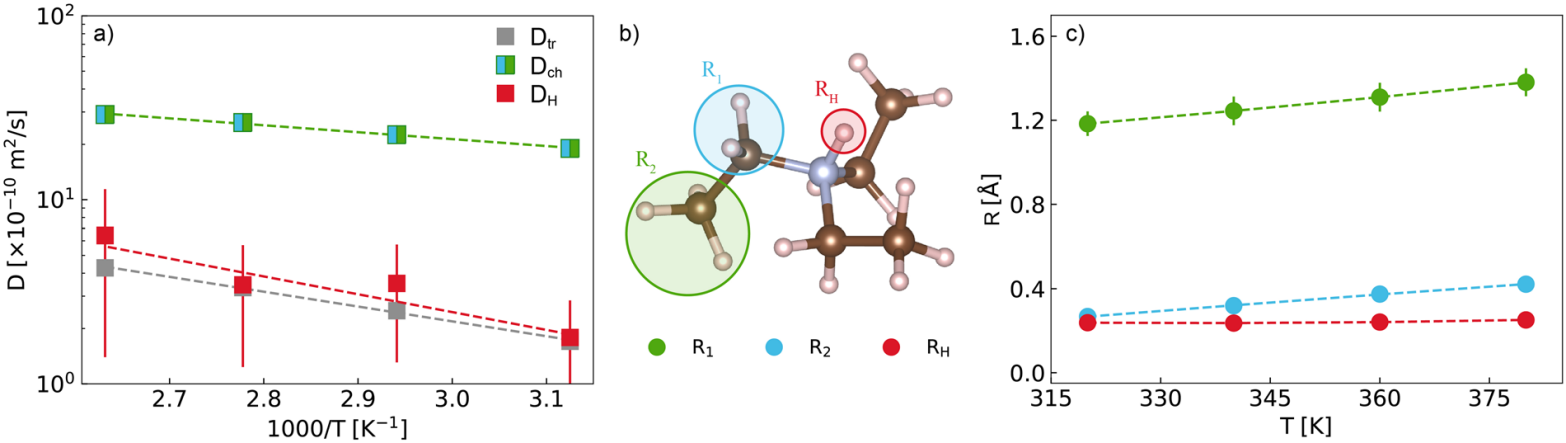
Temperature dependence of the parameters describing the cation single-particle dynamics in the liquid phase as obtained from the iterative fits of the TEA-TF and TEA_D_-TF spectra. (**a**) Self-diffusion coefficients of the long-range (global) diffusion, *D*_tr_, the localized motions of the N-H proton, *D*_H_, and the ethyl chains, *D*_ch_. The dashed lines are Arrhenius fits. (**b**) Sketch of the TEA structure and three groups of dynamically “equivalent” protons. (**c**) Temperature dependence of the confinement radii for the N-H proton (*R*_H_, red), bridging methylene (*R*_1_, blue), and terminal methyl (*R*_2_, green) groups. The dashed lines are guides to the eye.

As can be seen from Eq. 7 comparison between the completely protonated and paritally deuterated samples enables evaluation of a more complete set of parameters. The corresponding temperature dependencies are presented in Fig. 3. The long-range process of TEA-TF and TEA_D_-TF is characterized by the same value of the self-diffusion coefficient, *D*_tr_, suggesting that the isotope effect is minimal for the transport properties in the liquid state^14^. The obtained values of *D*_tr_ are in excellent agreement with the previously published data measured on the FOCUS time-of-flight spectrometer at SINQ, Switzerland with a similar resolution function^14^. The temperature dependence of *D*_tr_ follows the Arrhenius law $D={D}_{0}\,\exp \,(\,-\,{E}_{{\rm{A}}}/RT)$ with the activation energy ${E}_{{\rm{A}}}=15.4\pm 0.5$ kJ/mol. The existing quantitative and qualitative difference with the PFG-NMR results^26,27^ is the consequence of the significantly shorter time scale probed by QENS.

Various entangled localized motions of the ethyl chains are the source of the quasielastic component characterized by the parameters *R*_1_, *R*_2_, and *D*_ch_. After removing the coherent contribution, the diffusion coefficients turn out to be approximately twice as fast as the corresponding values obtained for the total, unseparated spectra on FOCUS^14^. Broader linewidths and consequently faster localized dynamics were also observed for the “pure” incoherent contribution of the aprotic pyridinium-based IL^22^. However, in the present case the effect is more pronounced indicating a greater impact of correlated dynamics of the three ethyl chains in the triethylammonium cation.

The restricted dynamics of the N-H proton discussed in our previous work^14^ are also present in the refined incoherent contribution of TEA_D_-TF, corroborating the earlier observations. The absolute values for *D*_H_ and *R*_H_ have yet changed significantly after the subtraction of the coherent component. To explain the nature of this process we addressed the DFT computations and MD simulations^28^. A close contact between the triethylammonium cation and the triflate anion in the gas-phase equilibrium corresponds to a strong hydrogen bond, the potential energy surface for the N-H proton exhibiting a single minimum. Thus, it is not highly probable that the observed localized process is related to a direct proton exchange between the cation and anion. In this regard, the MD simulations can offer some insights, because the N-H incoherent scattering function can be directly compared with those of the other atom groups. It should be mentioned, however, that the MD relaxation processes appear to be significantly slower as compared to the experiment. This a major drawback of non-polarizable force fields^28,38,39^. Moreover, while a sum of several exponents is a good approximation for the QENS intermediate scattering functions, the corresponding MD curves are decidedly “stretched”, requiring, for example, Kohlrausch-Williams-Watts (KWW) functions for modeling. Under these conditions only qualitative comparison between the MD simulations and the QENS data is possible.

**Figure 4:**
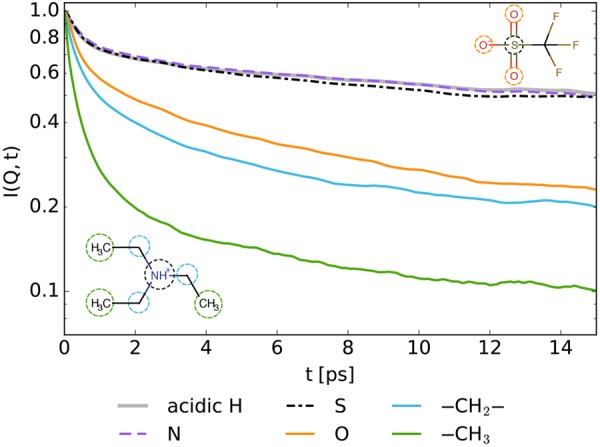
Intermediate scattering functions *I*(*Q*, *t*) at *Q* = 1.5 Å^−1^ as obtained from the MD trajectories. The relaxation curves of the N-H proton (gray solid line), the nitrogen atom of the triethylammonium cation (violet dashed line) and the sulfur of the triflate anion (black dash-dotted line) are practically identical on the picosecond time scale. The intermediate scattering functions of the bridging methelene (light blue solid line) and methyl (green solid line) hydrogens demonstrate a significantly faster decay due to localized motions of the ethyl chains and are presented for comparison. The relaxation of the oxygen intermediate scattering function (orange solid line) is also faster than that of the acidic H, N and S indicating that the breaking time of individual hydrogen bonds is shorter than the relaxation time of the ion pair correlated motion.

Figure 4 reveals a remarkable similarity between the intermediate scattering functions of the N-H proton, the nitrogen atom of the triethylammonium cation and the sulfur atom of the triflate anion during the first tens of picoseconds (the corresponding pair-correlation functions remain almost constant). This time-range is roughly equivalent to the experimental one including the discussed localized dynamics of the acidic proton. It means that the fast spatially restricted component of the TEA_D_-TF incoherent spectrum may reflect the localized dynamics of the anion-cation pair. According to the MD simulation, this correlated motion of the cation-anion pair persists over a longer time period than the lifetime of an individual hydrogen bond between them, as can be seen from a faster decay of the oxygen intermediate scattering function.

### Collective Dynamics

Although the difference between the H and D neutron coherent cross sections is less significant than that between the incoherent ones, the isotope substitution changes the sensitivity of the coherent QENS to different cross-correlation contributions (Fig. 2). Thus, ${S}_{{\rm{coh}}}(Q,E)$ of TEA_D_-TF is mainly affected by cation-cation inter- and intramolecular correlated motions, while the coherent spectrum of TEA-TF contains all components. The influence of collective cation-anion dynamics is most pronounced at the charge-charge diffraction peak (Q = 0.9 Å^−1^).

**Figure 5:**
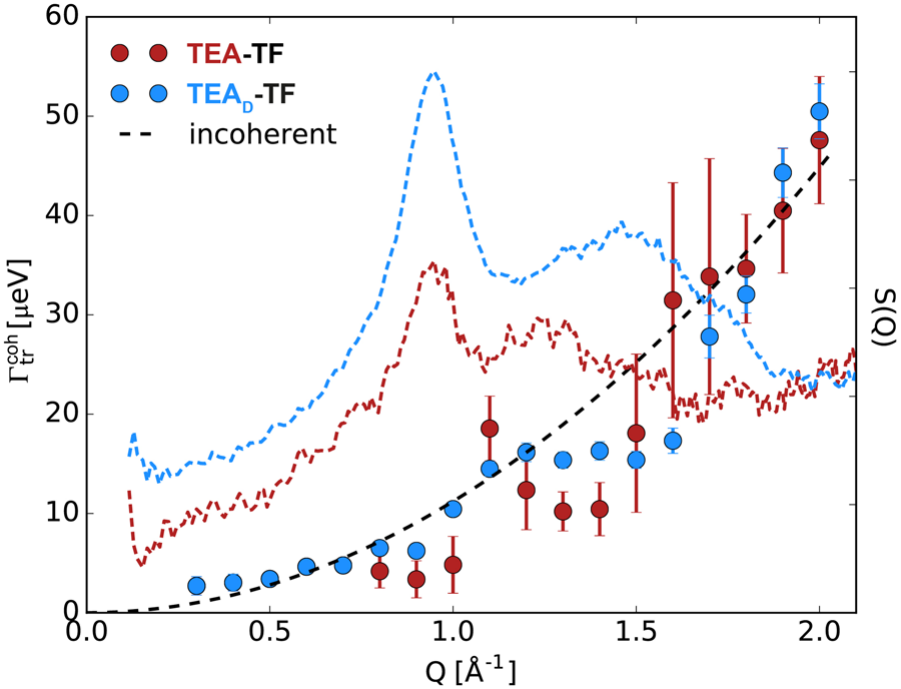
Linewidths of the narrow quasielastic contribution ${{\rm{\Gamma }}}_{{\rm{tr}}}^{{\rm{coh}}}$ of TEA-TF (red) and TEA_D_-TF (blue) as a function of *Q* at T = 320 K. Short-dashed color lines are the coherent contributions of the diffraction spectra, respectively. The black dashed line is the narrow quasielastic contribution of the incoherent spectrum calculated using Eq. 5.

In general two quasielastic contributions are required to describe the coherent spectra of both samples (Eq. 9, Fig. S8). The slower relaxation process is related to the long-range ion transport as can be seen from Fig. 5, where the linewidths ${{\rm{\Gamma }}}_{{\rm{tr}}}^{{\rm{coh}}}(Q)$ are compared with the Q-dependence calculated from the jump-diffusion model (Eq. 5) for the incoherent spectrum. The typical narrowing of the quasielastic lines at the diffraction correlation peaks, referred to as de Gennes narrowing^40^, can be seen for both TEA-TF and TEA_D_-TF. This effect literally means that there exist long-lived local arrangements of ions diffusing collectively on the picosecond time scale. The strength of the line modulation is comparable for both samples within experimental errors suggesting that the cat-cat, an-an, and cat-an cross-correlation diffusional components are characterized by similar or close relaxation times.

**Figure 6:**
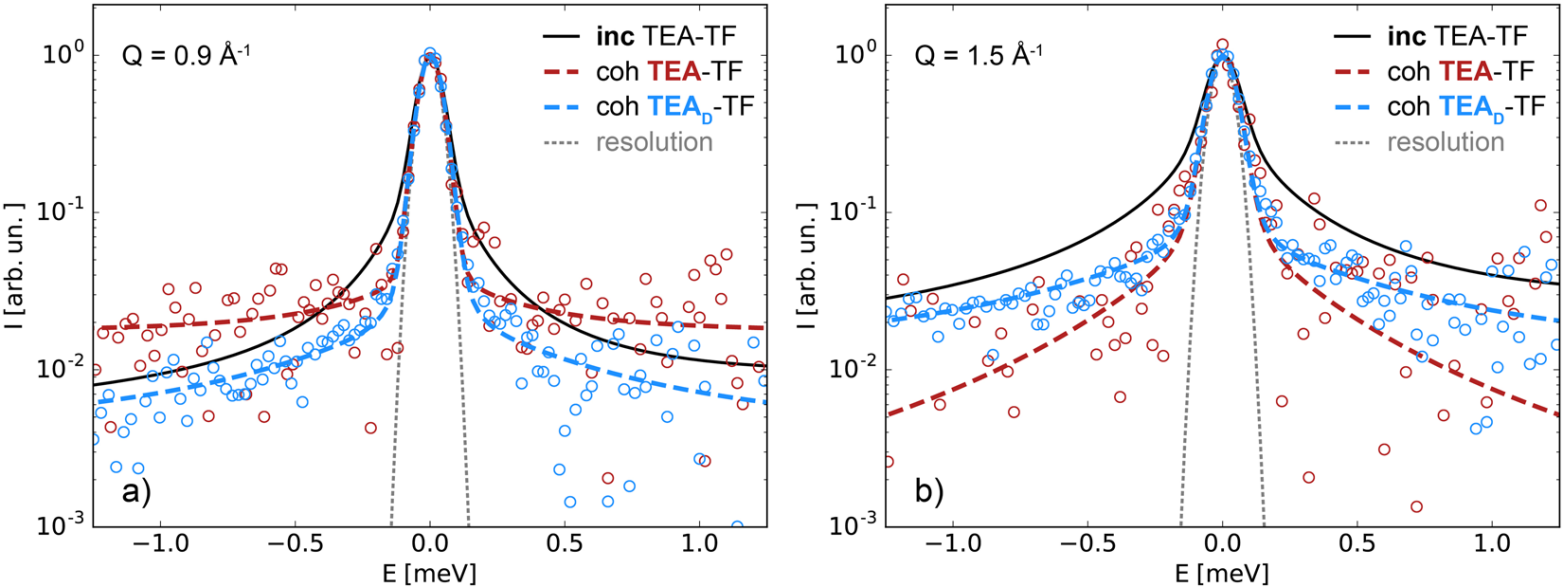
Coherent dynamic structure factor of TEA-TF (dashed red line) and TEA_D_-TF (dashed blue line) at the charge-charge (**a**) and adjacency (**b**) correlation peaks. The incoherent dynamic structure factor of the protonated sample (black solid line) is presented for comparison. The gray dotted line is the resolution linewidth at zero energy transfer for the D7 spectrometer.

In contrast to the previously studied case of the pyridinium-based IL^22^ ${S}_{{\rm{coh}}}(Q,E)$ of TEA-TF exhibits the second broader component, which allowed us to characterize the loss of coherence through localized dynamics. Fast intramolecular motions determine the shape of the QENS spectra in the energy transfer range of ~[−1, 1] meV (Fig. 6) and the observed effect strongly depends on the type of dominating correlations (cat-cat, cat-an, an-an), which can be highlighted by means of deuterium labeling. The dynamics of the ethyl chains (carbon and deuterium atoms) in TEA_D_-TF are mainly observed at the adjacency peak (1.3–1.8 Å^−1^). In this Q-range it is, therefore, possible to formally apply the Gaussian model as in the case of the incoherent spectra (Eq. 6). The estimates of the effective diffusion coefficient are very close to the *D*_ch_-values (Fig. 3a) and the characteristic confinement size is of order *R*_1_ (Fig. 3c). The quasielastic linewidths of the totally protonated sample are narrower due to the cat-an and an-an contributions, which slow down the average relaxation time. Thus, the coherence in local ion arrangements may be maintained despite fast stochastic intramolecular motions. This effect may arise from the stronger interaction between ions due to the hydrogen bond in the PIL, as well as it may depend on the ion size and shape. For example, in regard to internal dynamics the structures of the bis(trifluoromethylsulfonyl)imide anion and 1-butylpyridinum cation allow more degrees of freedom. As a result, the coherent contribution of this aprotic IL did not feature the second broader component^22^ seen in the present case. The charge-charge correlation peak at Q = 0.9 Å^−1^ corresponds to the length scale longer than that of the adjacency peak. It inevitably leads to a faster decay of the negative cat-an cross-correlation terms due to localized internal motions and, consequently, to a significantly broader QENS spectrum in the energy transfer range of 1 meV of TEA-TF as compared to TEA_D_-TF (Fig. 6a). Moreover, the observed difference may also originate from overall faster anion relaxation, because mainly the an-an component forms the charge-charge correlation peak of TEA-TF (Fig. 2).

## Conclusion

In summary, an extensive understanding of picosecond dynamics of the model protic ionic liquid TEA-TF has been achieved by means of a synergistic approach combining QENS with the polarization analysis and MD simulations. The experimental separation of coherent and nuclear spin-incoherent scattering permits a sophisticated description of collective and single-particle processes, while the MD analysis provides the tools to disentangle cross-correlation terms between selected groups of atoms.

Long-range diffusion as well as spatially restricted dynamics of the ethyl chains and the acidic proton have been characterized for the refined nuclear spin-incoherent spectra. Although the subtraction of the coherent contribution leads to significant changes in the estimates of the diffusion coefficients for localized dynamics, the qualitative picture of molecular motions seen with QENS on the picosecond time scale remains the same as has been previously inferred from the total dynamic structure factor. The enhanced localized dynamics of the acidic proton have been observed as well. Based on the analysis of the MD trajectories we assume that this process reflects the spatially restricted dynamics of cation-anion pairs in the liquid state.

The coherent QENS spectra have provided evidence of highly correlated picosecond motions in TEA-TF. The long-range diffusion can be considered as a collective process of ion associations. Their characteristic size is at least as large as the charge-charge periodicity of the PIL structure. Owing to the strong interaction between adjacent ions, the localized dynamics also turn out to be partially of collective nature, but motion coherence becomes gradually less significant at larger distances. Correlated long-range diffusion of ions on the picosecond time scale appears to be a common feature of both aprotic and protic ILs, whereas the nature of spatially restricted dynamics strongly depends on the ion structure and interaction between the ions. A complex interplay of single-particle and collective motions underlies the dynamical heterogeneity of ILs and accounts for their time scale-dependent transport characteristics.

## Materials and Methods

### Samples

The sample of TEA-TF ([NH(C_2_H_5_)_3_][SO_3_CF_3_]) and its partially deuterated analogue TEA_D_-TF ([NH(C_2_D_5_)_3_][SO_3_CF_3_]) were synthesized and characterized at the Department of Physical Chemistry, Saarland University (Sample Synthesis and NMR Characterization of the Supporting Information). The neutron scattering (coherent and incoherent) and absorption cross sections for the cation and for the anion are summarized in Table S1 of the Supporting Information.

### QENS experiment

QENS experiments^41^ in conjunction with polarization analysis were conducted on the D7 diffuse scattering spectrometer^30^ with the wavelength of incident neutrons of 5.7 Å covering the Q-values in the range of 0.3–2.0 Å^−1^. The measurements were performed both in the diffraction and time-of-flight modes. The polarization efficiency of the instrument was determined by measuring an amorphous quartz standard. The efficiency of the detectors was calibrated by measuring a vanadium standard. The frequency of the Fermi chopper used in the time-of-flight mode was equal to 145 Hz, providing the resolution function of 98 *μ*eV (FWHM)^42^. The low temperature incoherent TEA-TF spectra (T = 10 K) were used for the estimation of the linewidth of the instrument resolution. In order to minimize absorption and multiple scattering effects, an annular hollow cylindrical sample holder made of aluminum was used. The distance between the inner and outer cylinder was equal to 0.20 mm. Such a sample thickness guaranteed that neutron beam transmission through the sample exceeded 90%.

The standard data reduction of the D7 spectra was performed in the LAMP software package^43^. The raw data were corrected for empty cell, cryostat and time-independent (ambient neutrons/electronic noise) background contributions, sample geometry dependent self-attenuation and detector efficiency, converted to energy scale and finally binned into several Q-groups with ΔQ = 0.1 Å^−1^ to ensure adequate data statistics. After the separation of the coherent and nuclear spin-incoherent scattering^22,30^, simultaneous fitting in the (E, Q)-domain was performed in a program module^44^ based on the MPfit procedure^45^.

### MD analysis

The details of the MD simulation are presented in our previous publication^28^. The comparison of the MD trajectories with respect to the neutron scattering experiment was carried out using the nMoldyn/MDANSE software^31^. In particular, weighted incoherent and coherent intermediate scattering functions were calculated for all the particles in the simulation as well as for selected groups of atoms (bridging methylene groups, terminal methyl groups, N-H proton etc). The weights of the terms are defined from the neutron scattering lengths and proportional to ${b}_{\alpha ,{\rm{inc}}}^{2}$ and ${b}_{\alpha ,{\rm{coh}}}{b}_{\beta ,{\rm{coh}}}$ for the incoherent and coherent contributions, respectively.

## Electronic supplementary material

Supplementary Information

## Data Availability

The datasets generated and analysed during the current study are available from the corresponding author on request.
