# Supplementary material for: Linking Structure to Dynamics in Protic Ionic Liquids: A Neutron Scattering Study of Correlated and Single-Particle Motions
Source: Sci Rep. 2018 Nov 6;8:16400. doi: 10.1038/s41598-018-34481-w (PMC6219547; doi:10.1038/s41598-018-34481-w)
Supplement: Supplementary file 1 — Supplementary Information [file 41598_2018_34481_MOESM1_ESM.pdf]

Supporting Information: Linking Structure to Dynamics in Protic Ionic Liquids: A Neutron Scattering Study of Correlated and Single-Particle Motions

Tatsiana Burankova,*,† Juan F. Mora Cardozo,† Daniel Rauber,‡ Andrew Wildes,¶ and Jan P. Embs*,†

†*Laboratory for Neutron Scattering and Imaging, Paul Scherrer Institute, 5232 Villigen PSI, Switzerland*

‡*Department of Physical Chemistry, Saarland University, 66123 Saarbrücken, Germany*

¶*Institut Laue-Langevin, CS 20156, 38042 Grenoble Cedex 9, France*

E-mail: tatsiana.burankova@psi.ch; jan.embs@psi.ch

1 Sample Synthesis and NMR Characterization

Deuterated triethylamine-d^{15} (98% D) was delivered by Euriso-Top (Saarbrücken, Germany) and used immediately without further purification. Trifluoromethanesulfonic acid (⩾99%), triethylamine (⩾99%) DMSO-d^{6} (99.5% D) were purchased from Sigma Aldrich (St. Louis, USA). The water used purified using an Milli-Q® Type 1 ultrapure water system (Merck KGaA, Darmstadt, Germany). Synthesis of the ionic liquids was conducted in argon atmosphere. Identities of the synthesized ILs were confirmed by multinuclear NMR spectroscopy as well as ESI-MS. All samples were dried in oil pump vacuum (1×10^{-2} mbar) at 55 °C for three days prior to the measurements. NMR spectra were recorded on an AVANCE II 400 spectrometer (Bruker, Billerica, USA). Chemical shifts of the ^{1}H and ^{13}C{^{1}H} are given in ppm relative to tetramethylsilane using the residual solvent signal as reference. Chemical shifts of the ^{19}F{^{1}H} NMR spectra are given in relation to $CFCl_3$. The ESI-MS measurements of the ILs dissolved in acetonitrile were performed on a 2000 Qtrap Quadrupole Linear Ion Trap (AB Sciex, Darmstadt, Germany).

1.1 Synthesis of TEA-TF

To a solution of 5.89 mL trifluoromethanesulfonic acid (66.6 mmol; 1.0 eq.; 10.0 g) dissolved in 250 mL purified water under ice cooling were added 10.2 mL triethylamine (73.3 mmol; 1.1 eq; 7.42 g) dropwise. The homogenous, colorless solution was stirred for two hours at ambient temperature followed by the removal of water and excess of amine by means of rotary evaporation. The sample was dried on a vacuum line at 50 °C for two days with stirring to obtain 16.6 g of triethylammonium trifluoromethanesulfonate (66.1 mmol; 99% yield) as a colorless supercooled liquid.

^{1}**H NMR** (400 MHz, DMSO-d$_6$): δ = 8.83 (s, 1H, NH), 3.10 (qd, $^3J_{HH}$ = 7.2, $^3J_{HH}$ = 4.0 Hz, 6H, CH_2), 1.17 (t, $^3J_{HH}$ = 7.3 Hz, 9H, CH_3).
^{13}**C{^{1}H} NMR** (101 MHz, DMSO-d$_6$): δ = 120.69 (q, $^1J_{CF}$ = 322.3 Hz, CF_3), 45.78 (s, CH_2), 8.64 (s, CH_3).
^{19}**F{^{1}H} NMR** (376 MHz, DMSO-d$_6$): δ = -77.80 (s, CF_3).
ESI (+Q): m/z = 102 Da.
ESI (–Q): m/z = 149 Da.

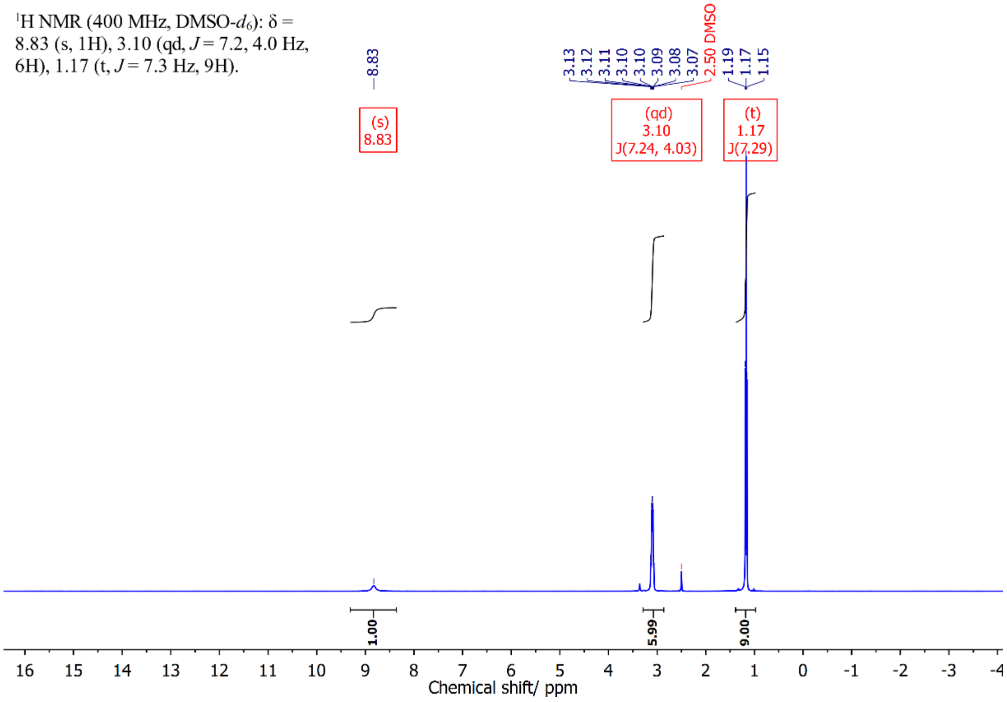

Figure S1: ^{1}H NMR spectrum of TEA-TF

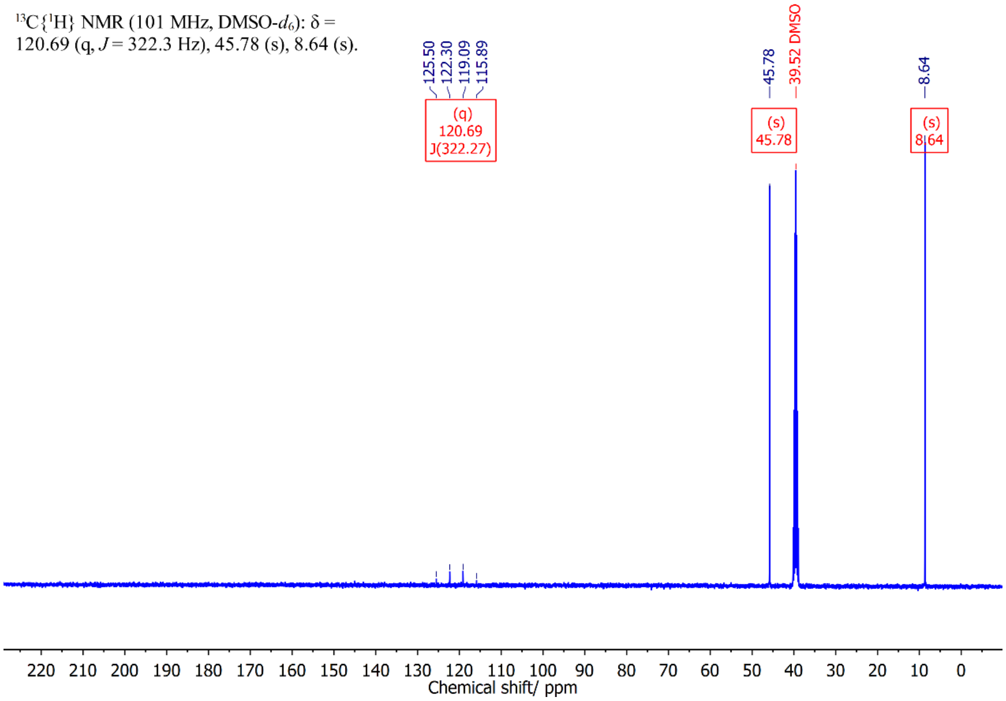

Figure S2: $^{13}C\{^{1}H\}$ NMR spectrum of TEA-TF

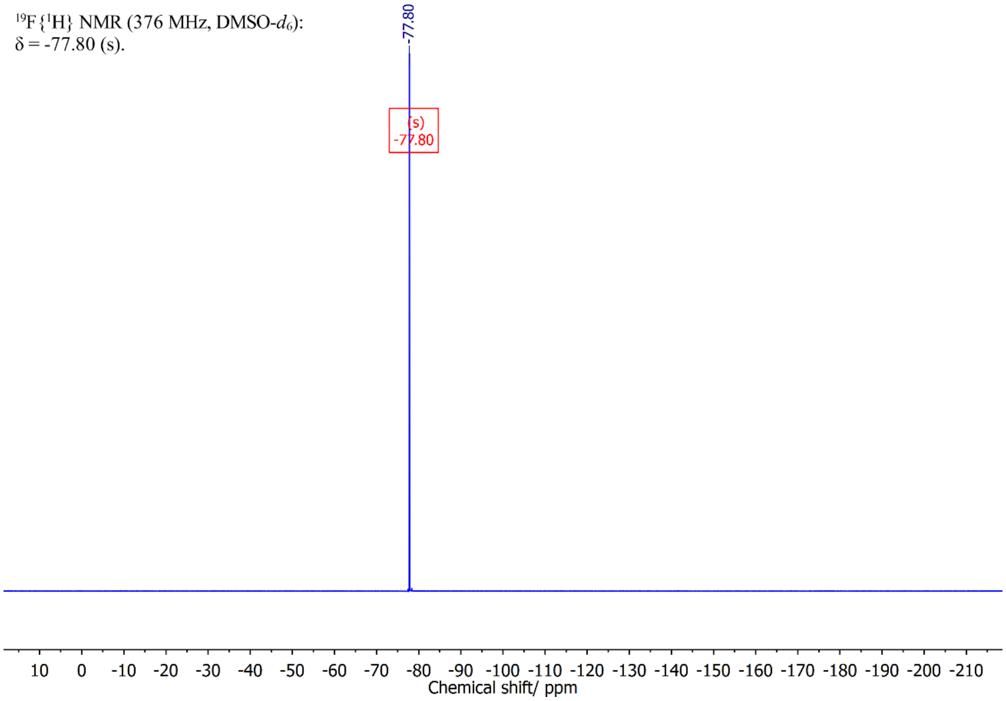

Figure S3: $^{19}F\{^{1}H\}$ NMR spectrum of TEA-TF

1.2 Synthesis of deuterated TEA_D-TF

2.07 mL of trifluoromethanesulfonic acid (23.5 mmol; 1.0 eq.; 3.53 g) were dissolved under ice cooling in 50 mL of purified water. To the ice cooled solution 4.1 ml d^{15}-triethylamine (25.8 mmol; 1.1 eq.; 3.00 g) were added dropwise. The ice bath was removed and the homogenous solution stirred for two hours at 25 °C. The water and slight excess of amine were removed on a rotary evaporator and the obtained product dried in oil pump vacuum at 50 °C for 48 hours. 6.18 g (23.3 mmol; 99% yield) of the alkyl-deuterated product were obtained as a colorless, supercooled liquid.

^{1}H NMR (400 MHz, DMSO-d_6): $\delta = 8.77$ (s, 1H, NH).
^{13}C{^{1}H} NMR(101 MHz, DMSO-d_6): $\delta = 120.62$ (q, $^{1}J_{CF} = 322.3$ Hz, CF_3), 45.54 – 44.15 (m, CD_2), 9.17 – 6.86 (m, CD_3).
^{19}F{^{1}H} NMR (376 MHz, DMSO-d_6): $\delta = -77.83$ (s, CF_3).
ESI (+Q): m/z = 117 Da.
ESI (–Q): m/z = 149 Da.

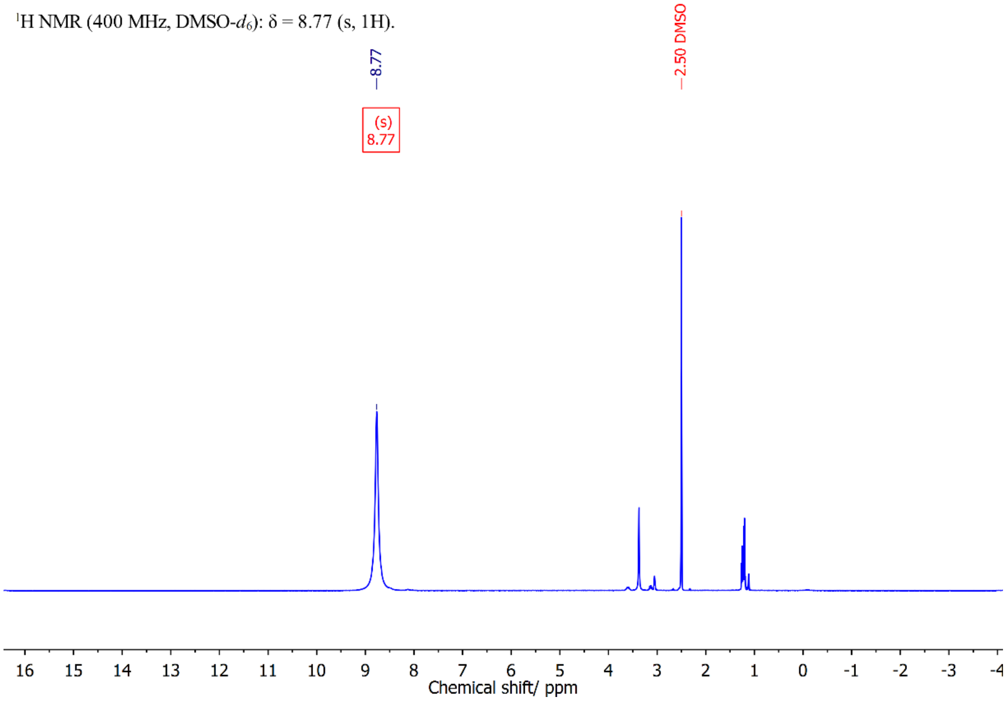

Figure S4: ^{1}H NMR spectrum of TEA_D-TF

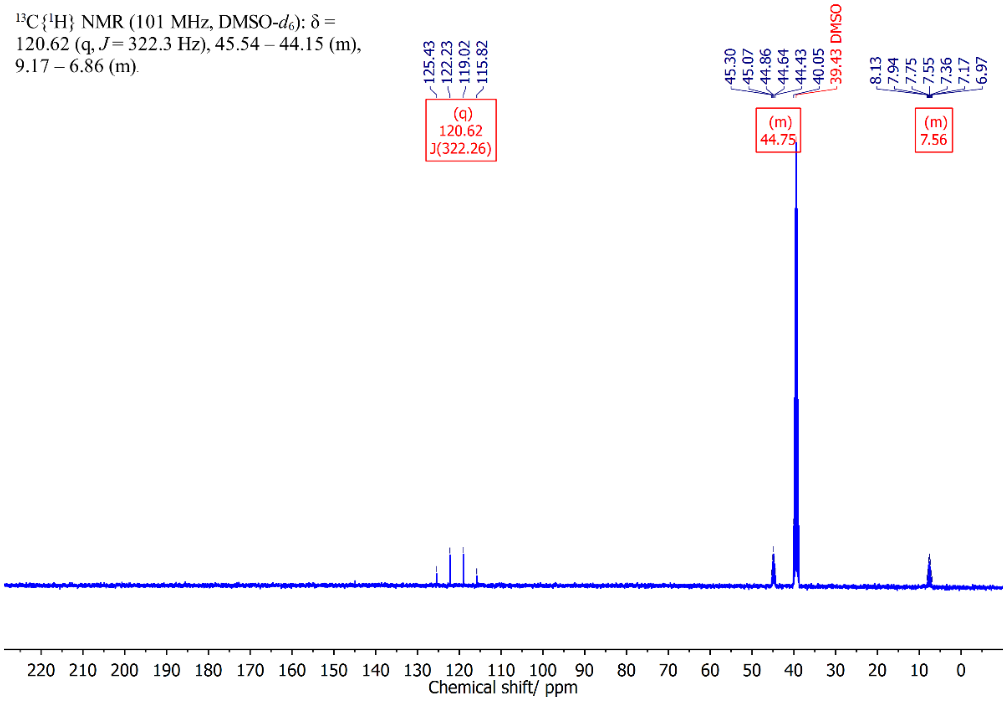

Figure S5: $^{13}C\{^{1}H\}$ NMR spectrum of TEA_{D}-TF

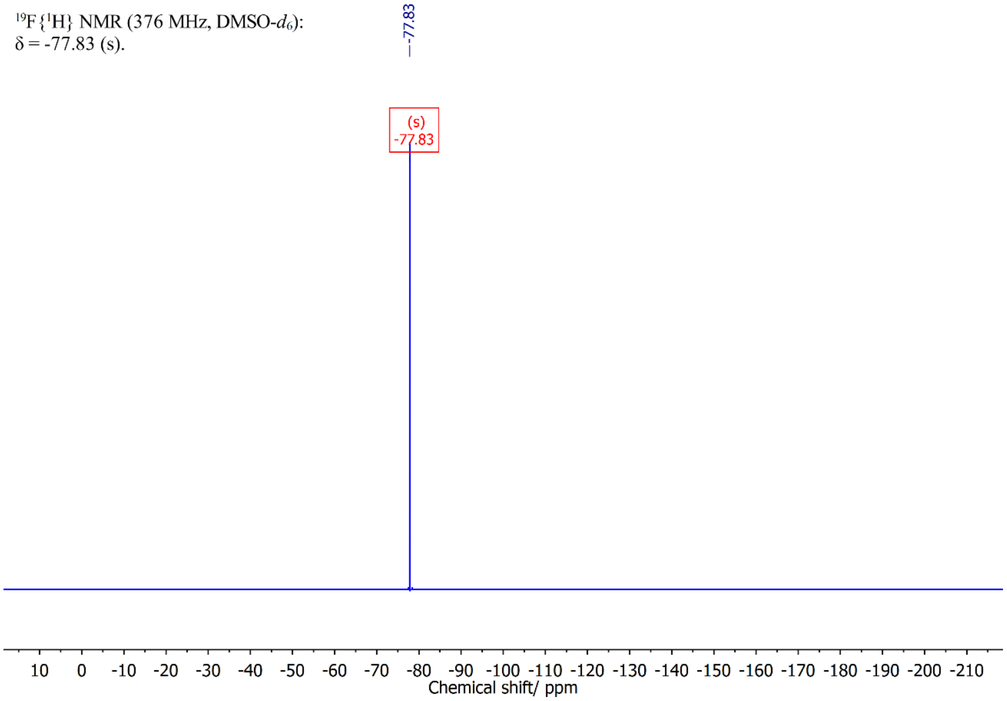

Figure S6: $^{19}F\{^{1}H\}$ NMR spectrum of TEA_{D}-TF

2 Neutron Scattering Cross Sections

Table S1: Summary of the Neutron Cross Sections[a]

System	σ_{scatt} [b]	σ_{abs} [b]	σ_{inc} [b]	σ_{coh} [b]	$\frac{\sigma_{\text{inc}}}{\sigma_{\text{scatt}}}$ [%]
H	82.02	1.05	80.26	1.76	0.98
D	7.64	0.00	2.05	5.59	0.27
TF	31.33	1.80	0.01	31.32	0.04
TEA	1357.27	23.16	1284.83	72.45	94.66
TEA-TF	1388.60	24.96	1284.84	103.77	92.53
TEA_{D}	241.48	7.23	111.53	129.95	46.19
TEA_{D}-TF	272.81	9.03	111.54	161.27	40.89

[a] σ_{abs} is given for neutrons with the wavelength of 5.70 Å. 1 b $= 10^{-28}$ m^2

3 Nuclear Spin-Incoherent Spectra

The model dynamic structure factor (eq 4) after making the appropriate substitution for $S_{\text{glob}}(Q,E)$ and $S_{\text{loc}}(Q,E)$ can be written in a general way as a sum of Lorentzian contributions, $\mathcal{L}(\Gamma,E)$

$$S_{\text{inc}}(Q,E) = \exp(-2W)\left\{ \overbrace{A_0(Q)\mathcal{L}(\Gamma_{\text{tr}},E)}^{A} + \overbrace{\sum_{n=1}^{\infty} a_{1,n}\mathcal{L}\left(\frac{\hbar n D_{\text{ch}}}{R_1^2} + \Gamma_{\text{tr}}, E\right)}^{B} \right. \\ \left. + \overbrace{\sum_{n=1}^{\infty} a_{2,n}\mathcal{L}\left(\frac{\hbar n D_{\text{ch}}}{R_2^2} + \Gamma_{\text{tr}}, E\right)}^{C} + \overbrace{\sum_{n=1}^{\infty} a_{\text{H},n}\mathcal{L}\left(\frac{\hbar n D_{\text{H}}}{R_{\text{H}}^2} + \Gamma_{\text{tr}}, E\right)}^{D} \right\} \tag{S1}$$

where term A accounts for the long-range diffusion of cations, terms B and C are the quasielastic contributions originating from the ethyl chains, term D appears due to the localized motion of the N-H proton.

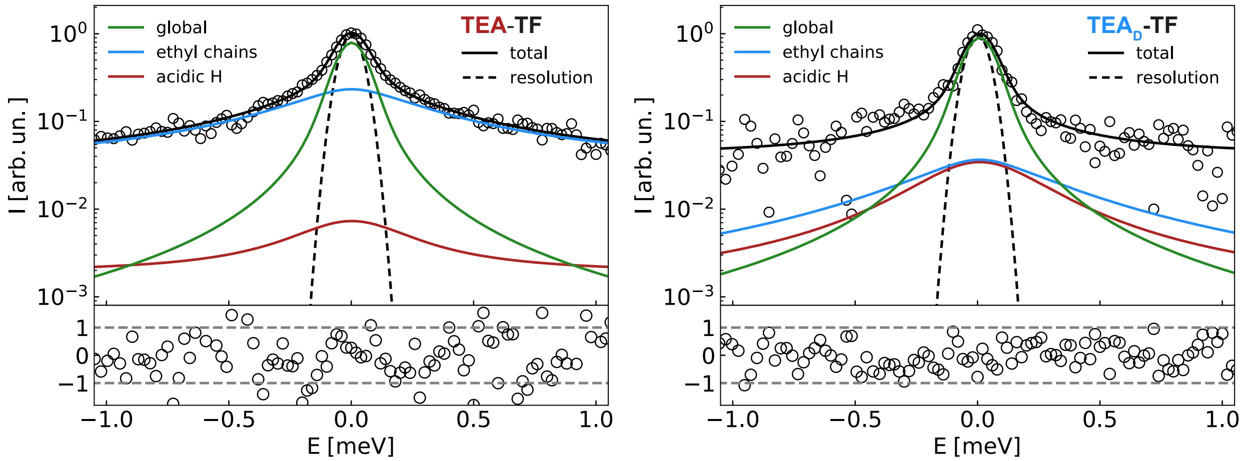

Figure S7: Incoherent spectra of TEA-TF and TEA$_D$-TF at T = 320 K and Q = 1.7 Å^{-1} with the model total fit functions (black solid line) and its Lorentizian subcomponents accounting for different dynamical contributions (see the text above for the explanation). Green line: long-range diffusion; blue line: quasielastic components originating from the ethyl chain dynamics; red line: the quasielastic component of the acidic proton. The relative contribution of the quasielastic terms is modulated through the site-selective H/D substitution and determined by the neutron incoherent cross section of H and D

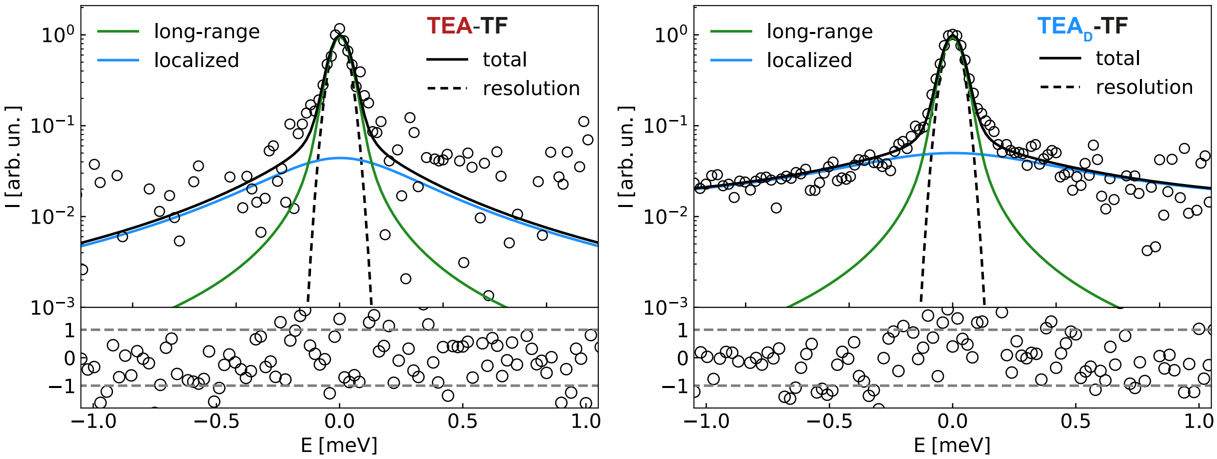

Figure S8: Coherent spectra of TEA-TF and TEA$_D$-TF at T = 320 K and Q = 1.5 Å^{-1} with the model total fit functions (black solid line) and its Lorentizian subcomponents accounting for long-range diffusion (green line) and localized dynamics (blue line)
